# Quality of Life and Respiratory Performance in the Laryngectomized Patient: Role of the HME Filters during Physical Activity

**DOI:** 10.3390/jcm13113137

**Published:** 2024-05-27

**Authors:** Massimo Mesolella, Salvatore Allosso, Mauro Mormile, Giuseppe Quaremba, Veronica Errante, Roberto D’Aniello, Giovanni Motta, Vincenzo Catalano, Gaetano Motta, Grazia Salerno

**Affiliations:** 1 Unit of Otorhinolaryngology, Department of Neuroscience, Reproductive Sciences and Dentistry, University Federico II of Naples, 80131 Naples, Italy; errante.veronika@gmail.com (V.E.); roberto.daniello@unina.it (R.D.); catalano.vincenzo1993@gmail.com (V.C.); grace@unina.it (G.S.); 2 Autonomic Service of Pneumology, Policlinical University Federico II, 80131 Naples, Italy; mauro.mormile@unina.it; 3 Department of Advanced Biomedical Sciences, Federico II University of Naples, 80131 Naples, Italy; quaremba@unina.it; 4 Unit of Otorhinolaryngology, Department of Mental and Physical Health and Preventive Medicine, University Luigi Vanvitelli, 80131 Naples, Italy; giovannimotta95@yahoo.com (G.M.); gaetano.motta@unicampania.it (G.M.)

**Keywords:** laryngeal cancer, total laryngectomy, rehabilitations, sport, HME filter, 6MWT

## Abstract

**Background:** Permanent tracheostomy because of total laryngectomy surgery entails significant consequences for patients regarding respiratory physiopathology, such as the loss of the filtering, humidifying, and heating of air by the nose. The use of special stomal filters can provide adequate protection of the tracheal–bronchopulmonary system with a reduction in respiratory pathologies. In fact, in most cases, laryngectomy patients are first cigarette smokers who for this reason also already have respiratory diseases such as chronic obstructive pulmonary disease (COPD). Despite the availability of tracheal filters, as reported in the literature, patients often tend to limit their use due to reported breathing difficulties, especially in conditions of intense breathing. **Methods:** The objective of this clinical study was to evaluate the most suitable stomal filter for laryngectomy patients during physical activity. The filters studied were an INHEALTH device (Blom-Singer SpeakFree HME); two ATOS devices (Provox^®^ Life™ Energy HME and Provox^®^ Life™ Home HME); and an FAHL device (Laryvox HME Sport). **Results**: For this purpose, the performances of 31 laryngectomy patients, subjected to medium–high physical effort, were analyzed through a standardized pneumological test, the Six Minute Walking Test (6MWT), which involves a sustained walk lasting six minutes, with an evaluation of heart rate, oxygen saturation, and meters traveled every 60 s; furthermore, we examined two subjective indices, namely, the basal and final dyspnea index and the initial and final muscular fatigue index. **Conclusions:** The multidisciplinary approach of the laryngectomee patient must also take pulmonary rehabilitation into consideration. It is the task of the medical team and speech therapy support to help the patient in the correct choice of HME filters taking into account daily needs.

## 1. Introduction

Laryngeal cancer represents 30% of head and neck cancers and 2% of malignant tumors. Despite progress in surgical and medical techniques, total laryngectomy is still today the operation of choice in the case of advanced forms of laryngeal cancer or in the case of salvage surgery [1,2,3,4,5,6].

The creation of a permanent stoma has profound psychological and physical consequences on the patient [7].

The loss of vocal ability is a devastating experience for the relational life of laryngectomy patients [8].

The need to breathe from the tracheostoma involves a series of problems, such as the loss of the heating, humidifying, and filtering function of the air by the nasal mucosa. This exposes the tracheobronchial tree of patients already compromised by smoking to recurrent respiratory infections. The loss of respiratory resistance caused by the larynx alters the normal functioning of the pulmonary alveoli, compromising gas exchange and causing a loss of smell due to loss of nasal breathing [9,10].

In a healthy subject, the air inspired from the external environment at a temperature of 22 °C and with a humidity of approximately 4% is heated at the level of the nasal passages, reaching 29 °C with a humidity that can reach 70%. Finally, at the level of the subglottic region, a further increase in temperature occurs, which reaches 32 °C and a humidity of 100%. In the small airways, the air temperature is the same as the body temperature. In laryngectomy patients, the air inspired by the tracheostoma reaches the lower airways at a temperature of 27–28 °C with a humidity of 50%. This has an important impact on the activity of the cilia of the respiratory system, which progressively reduce their movements until they remain immobile [11,12,13]. All this, combined with the lack of filtering action exerted by the nose, determines an increased risk of developing recurrent respiratory infections, an increase in coughing, and an increase in mucus production. These symptoms express themselves significantly in the first 6 months and then stabilize around 30 months after surgery. All these respiratory symptoms negatively affect fatigue, sleep quality, and social relationships. For this reason, in addition to respiratory rehabilitation, it is important that patients use heat and humidity exchangers (HMEs) early [14].

HME filters are also called artificial noses and have three fundamental characteristics: heat and humidity exchange capacity; resistance; particle filtering capacity.

The heat exchange occurs thanks to the retention of water by the filter. This is made up of a foam sponge treated with calcium salts and placed inside a plastic casing. This composition allows the air to be heated and water particles to be exchanged at the same time during breathing [15]. Furthermore, the stomal filter is capable of partially restoring the resistance offered by the larynx with an additional positive effect on the blowing noise produced at the stoma level, reducing it considerably [16]. The filtering capacity instead depends on the size of the pores that make up the spongy structure of the filter [17].

## 2. Materials and Methods

A prospective study was conducted on 31 consecutive patients who were enrolled at the U.O.C. of Otolaryngology of the A.O.U. Federico II of Naples from November 2023 to February 2024. All patients were informed regarding the methods, aims, and scope of this study.

In total, 27 men and 4 women aged between 41 and 80 (average 63 years) were recruited. All the patients enrolled had undergone phonatory rehabilitation using a tracheal–esophageal prosthesis: in 9 patients, it was inserted during the total laryngectomy operation, and in the others, it was inserted subsequently. The time since total laryngectomy was less than 3 years in 4 patients; between 3 and 5 years in 6 patients; greater than 5 years in 21 patients. A total of 26 patients were smokers before total laryngectomy; 18 patients used to consume alcoholic beverages. Out of 31 patients, 26 consistently used stomal filters. All patients stated that they carried out physical activity: 24 constantly, 7 occasionally.

The following were excluded from this study: patients with severe cardiac or bronchopulmonary pathologies; disease recurrence and ongoing adjuvant medical therapy.

**Figure 1:**
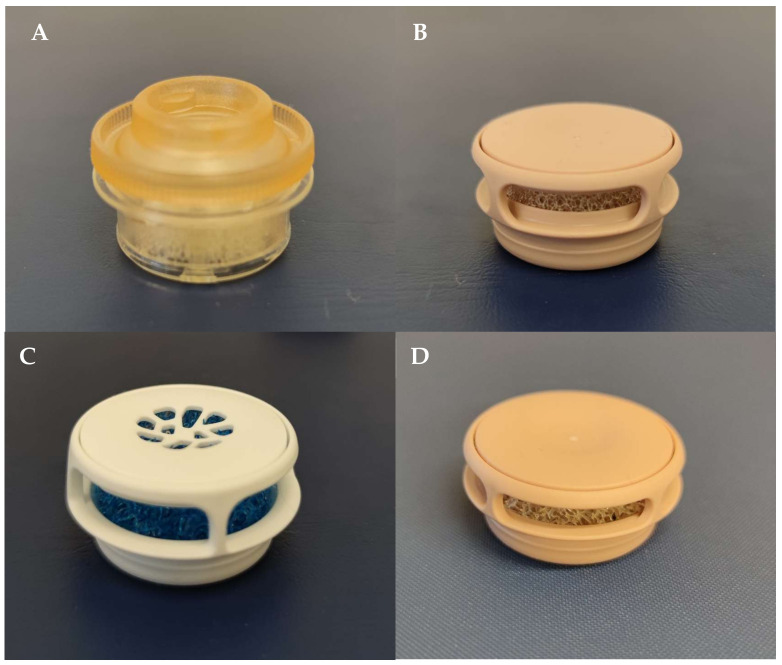
HME filters. (**A**) Bloom-Singer SpeakFree HME Hands Free Valve. (**B**) Laryvox HME Sport. (**C**) Provox^®^ Life^™^ Energy HME. (**D**) Provox^®^ Life^™^ Home HME.

The filters we tested in our study were as follows:

- Bloom-Singer SpeakFree HME Hands Free Valve (Figure 1A): Produced by the InHeath company (Buckinghamshire, UK), it is a system that does not require manual closure to speak, allowing hands-free phonation. It is an adjustable device, capable of adapting to the activity of the individual, who can choose between hands-free or digital occlusion. The filter with which the valve is equipped is the EasyFlow^®^ HME, which allows the subject to breathe more freely to satisfy their activity level and pulmonary needs.
- Laryvox HME Sport (Figure 1B): Produced by the Fahl company (Köln, Germany), designed to allow the practice of sport in laryngectomy patients and is useful in situations that require a greater need for air.
- Provox^®^ Life ™ Energy HME (Figure 1C): Produced by the Atos Medical company (West Allis, WI, USA), it provides good air humidification and low breathing resistance. It is designed for physically active individuals and features a diameter of 23 mm, slightly larger than its competitors. This increase in size is designed for optimal performance by ensuring the right balance between moisture-wicking, breathability, and size.
- Provox^®^ Life ™ Home HME (Figure 1D): Produced by the Atos Medical company, it offers the highest level of humidification compared to previous HMEs and is ideal for use at home or in activities that do not require deep breathing.

All enrolled patients underwent the Six Minute Walking Test (6MWT), which allows the simple and reliable measurement of the distance a person can walk in six minutes, walking as fast as possible on a flat surface [18,19].

Each patient performed the 6MWT with all four types of HME filters used in our experimentation, which were applied on the stomal adhesive in random succession while taking care that the type of filter was not recognized by the laryngectomy patient. The following parameters were evaluated before, during, and after the effort: blood oxygenation, heart rate, any dyspnea complained of, muscle fatigue, and distance traveled during the duration of the test.

Additionally, each patient gave a Borg scale value before and after the 6MWT [19]. The patient was invited to provide a value between 1 and 10 to express their respiratory and muscular fatigue, as this perception was considered an important element in the evaluation of physical performance together with the physiological measurements taken during the test.

This study was conducted in accordance with relevant guidelines and regulations. It was approved by the institutional review board committee of the Federico II University of Naples, Naples, Italy (2023/2092).

## 3. Results

### 3.1. Statistical Analysis

The data collected during the experimentation were examined using statistical analysis, in order to evaluate the presence of any significant differences between the four filters examined. The sample size was N = 31. For each numeric, sortable, and mutable variable, tables of absolute frequencies, relative percentages, and cumulative percentages were created. Additionally, means and standard deviations were determined for each variable. Any differences observed between the means of each variable for dependent samples were determined through the one-way ANOVA procedure, the Bonferroni multiple test, the test of the homogeneity of variances through Levene’s statistics, and Dunnett’s T3 test to test the possible homoscedasticity of variances. Significance was set equal to 0.05 and 95% confidence intervals were determined. The bivariate correlation matrix was calculated.

To verify the presence of any significant correlations, the linear correlation coefficient was determined according to Pearson, complete with the one-tailed (with the level of sig. = 0.05) and two-tailed (with the level of sig. = 0.01) significance tests. To facilitate interpretation, diagrams of the regression line interpolating the observed data were produced.

The processing was carried out with the multifactorial and multidimensional statistical analysis program IBM SPSS statistics, ver. 28.0.1.1.

### 3.2. Data Interpretation

The parameters recorded for each individual patient included instrumental data, therefore, objective (saturation, heart rate, meters traveled) and subjective data (basal and final dyspnea index and basal and final fatigue index), the latter being the result of the patient’s subjective perception and measured referring to the Borg CR10 scale.

By using the saturation parameter as the dependent variable, a multiple comparison was carried out between the four filters studied. As can be seen from the Bonferroni test (Table 1) in terms of saturation, there is a significant difference between the Provox^®^ Life™ Home HME filter and the Blom-Singer SpeakFree HME filter, and between the Blom-Singer SpeakFree HME filter and the Laryvox HME Sport filter. The Provox^®^ Life™ Energy HME filter does not show significant differences with the other filters considered.

A major correction was carried out with the Dunnet test, which assumes that the variances are not equal; nevertheless, what has just been described was verified (Table 1).

**Figure 2:**
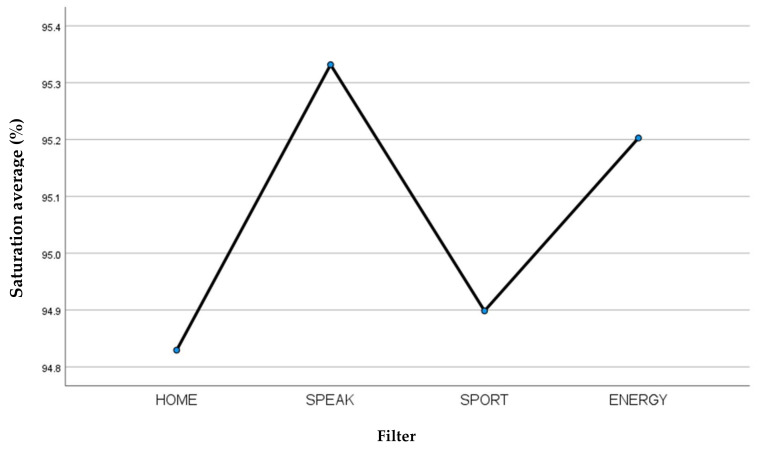
Average saturation recorded for each filter.

Considering the average of the saturation parameter (Table 2), it can be seen that the Blom-Singer SpeakFree HME filter was the best performing with respect to the parameter considered (Figure 2).

**Figure 3:**
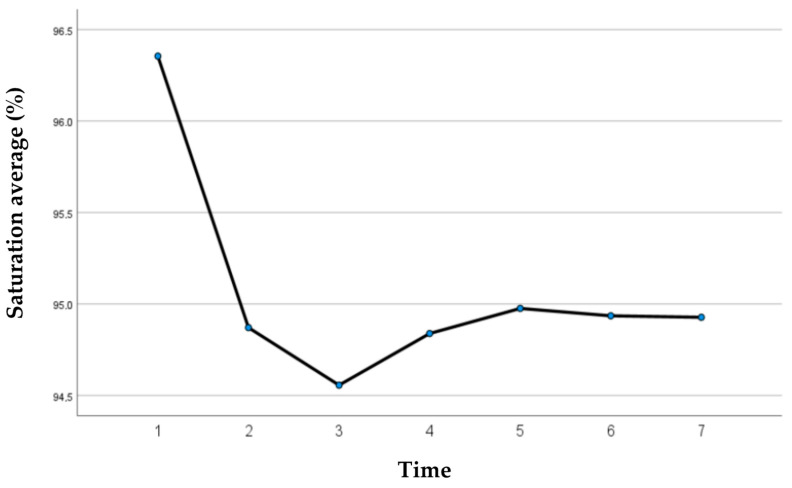
Saturation averages’ variation over time.

The saturation values were studied in the six minutes covered by the test to evaluate whether there were significant differences during the six minutes; a significant difference was observed only in the first minute (Figure 3).

Regarding the study of heart rate (HR) as a dependent variable in the multiple comparisons between the four filters studied, the Bonferroni test was applied, which did not find any significant differences (Table 3).

**Figure 4:**
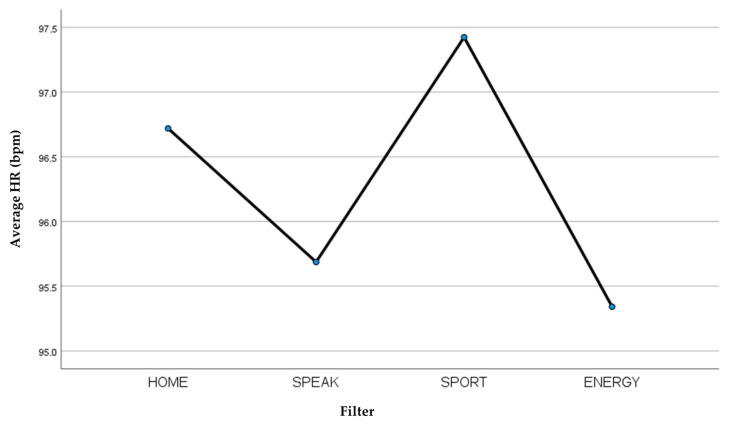
Average HR recorded for each filter.

If we consider the average of the HR parameter (Table 4), from a graphic point of view, we can observe a higher heart rate with the Laryvox HME Sport filter and a lower heart rate with the Provox^®^ Life™ Energy HME filter, but this difference is not statistically significant (Figure 4).

Then, the parameter of meters traveled was examined. The Bonferroni test was applied in the multiple comparisons between the four filters studied and it was set as a variable depending on the meters traveled (Table 5); the significant differences found were the following:

- The Provox^®^ Life™ Home HME filter showed a significant difference compared to the other three filters;
- The Laryvox HME Sport filter showed a significant difference compared to the Provox^®^ Life™ Home HME and Blom-Singer SpeakFree HME filters;
- The Blom-Singer SpeakFree HME filter showed significant differences compared to the Provox^®^ Life™ Home HME and Laryvox HME Sport filters;
- The Provox^®^ Life™ Energy HME filter showed a significant difference only compared to the Provox^®^ Life™ Home HME filter.

The Dunnet test confirms the Bonferroni test (Table 5).

**Figure 5:**
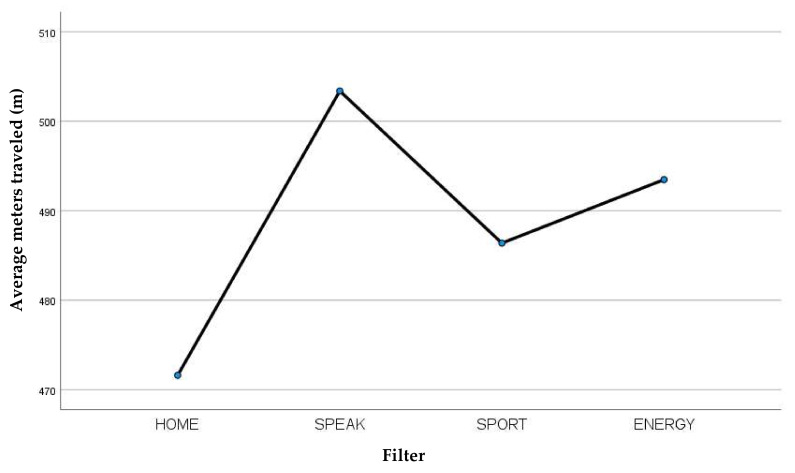
Average meters traveled recorded for each filter.

Furthermore, considering the average meters traveled during the 6MWT (Table 6) we observe that the Blom-Singer SpeakFree HME filter was the best performing with respect to the parameter considered (Figure 5).

As regards the subjective parameters, placing the final subjective dyspnea index as the dependent variable in the multiple comparisons (Table 7) between the four filters examined, a significant difference was found between the following:

- The Provox^®^ Life™ Home HME filter and Blom-Singer SpeakFree HME filter;
- The Provox^®^ Life™ Home HME filter and Provox^®^ Life™ Energy HME filter;
- The Blom-Singer SpeakFree HME filter and Provox^®^ Life™ Energy HME filter;
- The Laryvox HME Sport filter and Provox^®^ Life™ Energy HME filter.

The Dunnet test confirms the Bonferroni test (Table 7).

**Figure 6:**
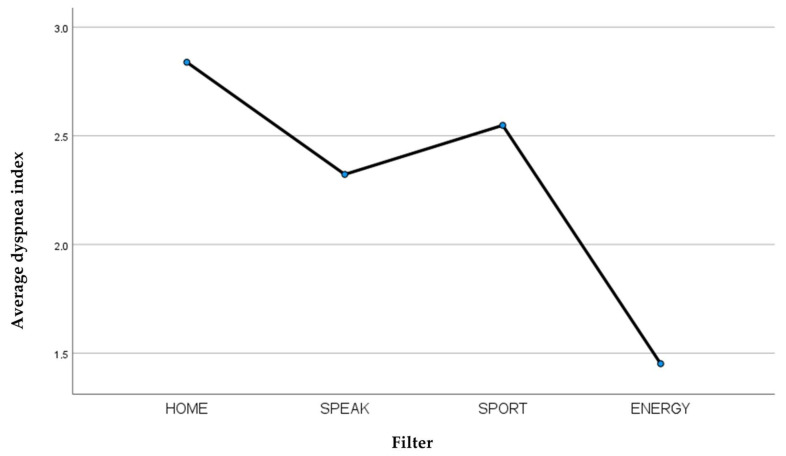
Average dyspnea index recorded for each filter.

Comparing the averages of the final dyspnea index of the four filters examined (Table 8), it is observed that the Provox^®^ Life™ Energy HME filter was the one best tolerated by patients in the physical effort exerted during the execution of the SMWT (Figure 6).

By placing the final subjective fatigue index as the dependent variable in the multiple comparisons (Table 9) between the four filters examined, a significant difference was found between the following:

- The Provox^®^ Life™ Home HME filter and Blom-Singer SpeakFree HME filter.
- The Provox^®^ Life™ Home HME filter and Provox^®^ Life™ Energy HME filter.
- The Blom-Singer SpeakFree HME filter and Provox^®^ Life™ Home HME filter.
- The Blom-Singer SpeakFree HME filter and Provox^®^ Life™ Energy HME filter.
- The Laryvox HME Sport filter and Provox^®^ Life™ Energy HME filter.
- The Provox^®^ Life™ Energy HME filter and Provox^®^ Life™ Home HME filter.
- The Provox^®^ Life™ Energy HME filter and Blom-Singer SpeakFree HME filter.
- The Provox^®^ Life™ Energy HME filter and Laryvox HME Sport filter.

The Dunnet test confirms the Bonferroni test (Table 9).

**Figure 7:**
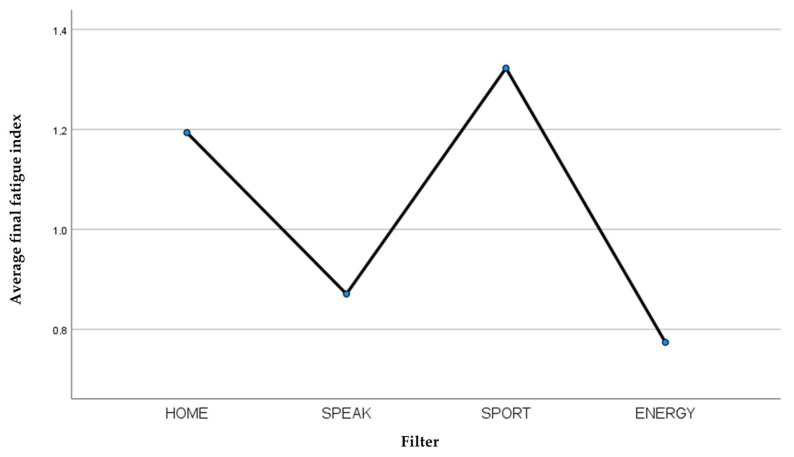
Average final fatigue index recorded for each filter.

Comparing the averages of the final fatigue index of the four filters examined (Table 10), it is observed that the Laryvox HME Sport filter was the least tolerated, while the Provox^®^ Life™ Energy HME filter was the most tolerated by patients in the physical effort exerted during the execution of the 6MWT (Figure 7).

**Figure 8:**
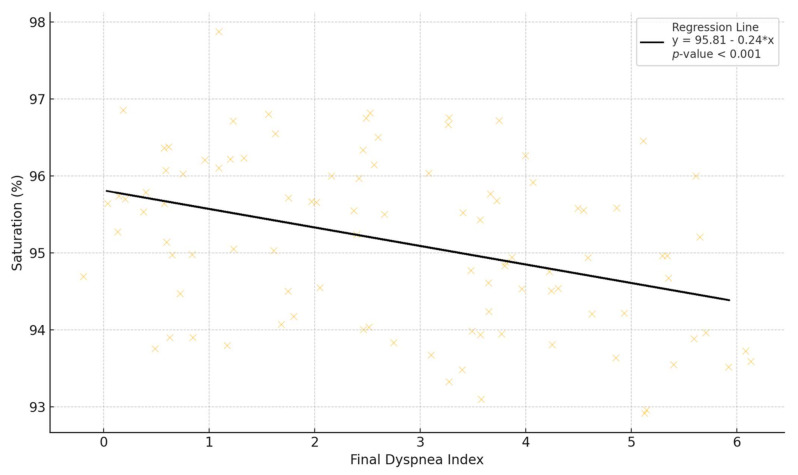
Diagram of the regression line interpolating the saturation data and final dyspnea index.

Upon completion of the investigation, we wanted to evaluate whether there was a statistically significant correlation between the instrumental and subjective data. The following can be seen from Table 11:

- Saturation has a significant inverse correlation at the 0.01 level with the baseline dyspnea index; this means that subjects with a reported perception of dyspnea at the start recorded lower saturation values during the execution of the 6MWT;
- The meters traveled have a significant inverse correlation at the 0.01 level with the basal dyspnea index, i.e., the subjects who reported some dyspnea at the start walked fewer meters during the test;
- The final dyspnea index presented a significant inverse correlation at the 0.01 level with saturation (Figure 8) and with meters traveled and a significant direct correlation at the 0.01 level with heart rate; this means that the subjects who reported higher values of perceived dyspnea after the 6 min of testing recorded, in the instrumental data, lower saturation values and lower number of meters traveled, whereas the heart rate had higher values;
- Final work showed a significant inverse correlation at the 0.05 level with saturation and a significant inverse correlation at the 0.01 level with heart rate; therefore, subjects who reported relevant tiredness after carrying out the test recorded lower saturation values and fewer meters traveled.

## 4. Discussion

The use of HME stomal filters in laryngectomy patients represents a fundamental pulmonary rehabilitation method that allows the maintenance of respiratory system physiology as close as possible to that existing before the surgery, considerably reducing the incidence of inflammatory pathologies, even severe ones [12,15,20,21]. The prevention of respiratory complications is also of great importance during vocal rehabilitation, especially in cases of the use of a tracheal–esophageal prosthesis; the exhaled pulmonary air as an air current sets the pseudo glottis in vibration; therefore, good pulmonary performance, with reduced tracheal–bronchial secretion, is closely related to satisfactory voice quality [22,23,24].

The process of social reintegration of laryngectomy patients begins with vocal rehabilitation: as the voice is an essential tool in human life, especially in the relational sphere, even when the vocal function is returned, sometimes, no attention is paid to welfare and the importance of having an active lifestyle for social reintegration [8,25,26,27]. Sport, among other things, represents a vehicle for social inclusion, an important tool for aggregation and interaction that, especially for these patients, is capable of distancing them from the state of anguish resulting from their illness and at the same time allowing them to feel socially accepted [6,28,29,30].

Over the years, various models of HME filters have been proposed as devices that allow both adequate protection of the tracheal–bronchial tree and respiratory resistance suitable for the physical activities carried out by the patient to satisfy the various needs of daily life [31]. The main problems were related to the creation of a filter capable of allowing the practice of more intense motor activities, such as those associated with sport, considering that, unfortunately, many patients are mostly part of a younger age group [17,22]. An HME filter suitable for sports practice must have less resistance to air flow; currently, the most used ones are the following:

- Provox^®^ Life™ Go HME;
- Laryvox HME Sport;
- Provox^®^ Life™ Energy HME;
- Provox^®^ XtraFlow HME™;
- Blom-Singer EasyFlow HME;
- Blom-Singer SpeakFree HME;
- Laryvox^®^ Extra HME.

The data obtained in our study reveal that with regard to the objective parameters measured during the 6MWT, the best results, which were also statistically significant, were obtained with the Blom-Singer SpeakFree HME filter, despite the Provox^®^ Life™ Energy HME filter receiving the widest approval from patients when we evaluated the final dyspnea index.

These results lead to several considerations; first of all, this is a preliminary study, with a limited series of cases and with an instrumental evaluation conducted for a short period of time, a condition that can obviously be different from what the patient experiences during the practice of their usual physical activities (e.g., cycling, walking, gym, Pilates, etc.).

The Blom-Singer SpeakFree HME filter and the Provox^®^ Life™ Energy HME filter can be considered equivalent in daily practice and certainly much more suitable for more intense physical activity than traditional filters; however, it is necessary to plan studies that evaluate the same parameters we used in a longer period of motor activity, in order to better define the respiratory resistance characteristics perceived by the patients and compare them with the results obtained by measuring saturation and heart rate.

It would be useful in a future study to expand the sample under examination and test the differences between the parameters considered for different types of physical activity; for example, we could test physical activity by means of a test on an exercise bike for a longer period than 6 min. We also remember that all the possible tests do not take into account some important characteristics such as sweating and the different climatic conditions that the patient may encounter in real life and that can compromise the validity of the HME filter.

## 5. Conclusions

Patients undergoing total laryngectomy inevitably experience significant changes in their quality of life, not only due to anatomical and functional variations, which limit the performance of numerous activities, but above all due to the psychological impact that the oncological pathology and these limitations have on the subject [7,25,32]. The resulting repercussions concern a vast range of aspects; the main problem undoubtedly concerns the area of verbal communication, but there are also food problems, more than anything else, which can be traced back to a reduction in the senses of taste and smell, which determine a lower appreciation of food [8,33]. Furthermore, there is a decrease in strength and physical resistance, which leads to difficulty in carrying out strenuous activities and, in more serious cases, even simple daily activities. Concern about one’s physical appearance and one’s voice is what most affects the psychological well-being of laryngectomy patients, leading them to maintain a distance from the world around them and to withdraw into themselves, thinking that other people find them unpleasant [34,35].

Consequently, although laryngeal cancer has a good cure rate, it is equally true that it disturbs patients’ psychological balance throughout their lives, influencing their habits and constantly reminding them of the cancer experience, due to the permanent presence of the tracheostoma [20,32,36]. Considering this, it is wise to take note of the change in the quality of life of laryngectomy patients, but, at the same time, also of the current therapeutic and rehabilitative supports, which allow the patients to compensate for this handicap [6,37]. In fact, restoring the patients to a quality of life as similar as possible to the pre-operative one represents an essential objective in the rehabilitation field; unfortunately, it does not seem adequately considered, with the relationship with the laryngectomy very often focusing only on the oncological and vicarious vocal aspects [36,38].

It is the task of the speech therapist, together with the doctor, to illustrate the various aids for the treatment of tracheal stomas and the importance of using HME filters due to the enormous advantages they provide at a pulmonary and relational level [20]. Even today, many laryngectomies do not use stomal filters, and this can essentially be attributed to a lack of information received; therefore, it is the primary task of the healthcare team to inform patients, both pre-operatively and subsequently, of the possibilities that modern technologies offer for the best management of tracheostomas [15,22].

In our study, both Blom-Singer SpeakFree HME and Provox^®^ Life™ Energy HME proved to be the most suitable filters for patients’ physical performance during testing, the first regarding the instrumental data of better saturation, reduced heart rate values, and greater number of meters traveled, while the second one was more appreciated by the patients due to their perception of less dyspnea and fatigue during the test. Whatever the patient’s choice, the important thing is that an HME filter is always used as it will guarantee the patient a better physical condition and the possibility of returning quickly and satisfactorily to previous activities, even the most demanding ones, when they accept their new anatomical–physiological condition with much more serenity.

## Figures and Tables

**Table 1:** Multiple comparisons. Dependent variable: saturation.

	(I) Filter	(J) Filter	Mean Difference (I − J)	SE	Significance	Confidence Interval 95%	
Bonferroni	HOME	SPEAK	−0.50 *	0.14	0.003	−0.88 to	−0.12
		SPORT	−0.07	0.14	1.000	−0.45 to	0.31
		ENERGY	−0.37	0.14	0.058	−0.75 to	0.01
	SPEAK	HOME	0.50 *	0.14	0.003	0.12 to	0.88
		SPORT	0.43 *	0.14	0.016	0.05 to	0.81
		ENERGY	0.13	0.14	1.000	−0.25 to	0.51
	SPORT	HOME	0.07	0.14	1.000	−0.31 to	0.45
		SPEAK	−0.43 *	0.14	0.016	−0.81 to	−0.05
		ENERGY	−0.30	0.14	0.210	−0.69 to	0.08
	ENERGY	HOME	0.37	0.14	0.058	−0.01 to	0.75
		SPEAK	−0.13	0.14	1.000	−0.51 to	0.25
		SPORT	0.30	0.14	0.210	−0.08 to	0.69
T3 Dunnett	HOME	SPEAK	−0.50 *	0.14	0.002	−0.87 to	−0.13
		SPORT	−0.07	0.15	0.998	−0.46 to	0.32
		ENERGY	−0.37	0.16	0.100	−0.79 to	0.04
	SPEAK	HOME	0.50 *	0.14	0.002	0.13 to	0.87
		SPORT	0.43 *	0.13	0.006	0.09 to	0.78
		ENERGY	0.13	0.14	0.927	−0.24 to	0.50
	SPORT	HOME	0.07	0.15	0.998	−0.32 to	0.46
		SPEAK	−0.43 *	0.13	0.006	−0.78 to	−0.09
		ENERGY	−0.30	0.15	0.223	−0.70 to	0.09
	ENERGY	HOME	0.37	0.16	0.100	−0.04 to	0.79
		SPEAK	−0.13	0.14	0.927	−0.50 to	0.24
		SPORT	0.30	0.15	0.223	−0.09 to	0.70

* Mean difference is significant at 0.05. In bold: statistically significant values.

**Table 2:** Preliminary summary statistics relating to saturation data.

		*n*	Mean	SD	SE	Middle Confidence Interval 95%		Min	Max	Components Variance
HOME		217	**94.83**	1.63	0.111	94.61 to	95.05	91	98	
SPEAK		217	**95.33**	1.25	0.085	95.16 to	95.50	92	99	
SPORT		217	**94.90**	1.46	0.099	94.70 to	95.09	91	98	
ENERGY		217	**95.20**	1.63	0.111	94.98 to	95.42	90	99	
Total		868	**95.07**	1.51	0.051	94.96 to	95.17	90	99	
Model	Fixed effects			1.50	0.051	94.97 to	95.17			
	Casual effects				0.120	94.68 to	95.45			0.047

Note: N = 31 people × 7 times = 217. In bold: statistically significant values.

**Table 3:** Multiple comparisons. Dependent variable: heart rate (HR).

	(I) Filter	(J) Filter	Mean Difference (I − J)	SE	Significance	Confidence Interval 95%	
Bonferroni	HOME	SPEAK	1.03	0.86	1.000	−1.25 to	3.31
		SPORT	−0.70	0.86	1.000	−2.99 to	1.58
		ENERGY	1.38	0.86	0.663	−0.90 to	3.66
	SPEAK	HOME	−1.03	0.86	1.000	−3.31 to	1.25
		SPORT	−1.74	0.86	0.266	−4.02 to	0.54
		ENERGY	0.35	0.86	1.000	−1.94 to	2.63
	SPORT	HOME	0.70	0.86	1.000	−1.58 to	2.99
		SPEAK	1.74	0.86	0.266	−0.54 to	4.02
		ENERGY	2.08	0.86	0.096	−0.20 to	4.36
	ENERGY	HOME	−1.38	0.86	0.663	−3.66 to	0.90
		SPEAK	−0.35	0.86	1.000	−2.63 to	1.94
		SPORT	−2.08	0.86	0.096	−4.36 to	0.20
T3 Dunnett	HOME	SPEAK	1.03	0.89	0.816	−1.32 to	3.38
		SPORT	−0.70	0.88	0.963	−3.03 to	1.62
		ENERGY	1.38	0.84	0.479	−0.85 to	3.61
	SPEAK	HOME	−1.03	0.89	0.816	−3.38 to	1.32
		SPORT	−1.74	0.88	0.260	−4.06 to	0.59
		ENERGY	0.35	0.85	0.009	−1.89 to	2.58
	SPORT	HOME	0.70	0.88	0.96	−1.62 to	3.03
		SPEAK	1.74	0.88	0.26	−0.59 to	4.06
		ENERGY	2.08	0.83	0.07	−0.12 to	4.29
	ENERGY	HOME	−1.38	0.84	0.48	−3.61 to	0.85
		SPEAK	−0.35	0.85	1.00	−2.58 to	1.89
		SPORT	−2.08	0.83	0.07	−4.29 to	0.12

**Table 4:** Preliminary summary statistics relating to HR.

		N	Mean	SD	SE	Middle Confidence Interval 95%		Min	Max	Components Variance
HOME		217	**96.72**	9.25	0.628	95.48 to	97.96	70	123	
SPEAK		217	**95.69**	9.29	0.630	94.44 to	96.93	60	116	
SPORT		217	**97.42**	9.06	0.615	96.21 to	98.64	60	113	
ENERGY		217	**95.34**	8.31	0.564	94.23 to	96.45	66	118	
Total		868	**96.29**	9.01	0.306	95.69 to	96.89	60	123	
Model	Fixed effects			8.98	0.305	95.69 to	96.89			
	Casual effects				0.477	94.77 to	97.81			0.539

Bold: statistically significant values.

**Table 5:** Multiple comparisons. Dependent variable: meters traveled.

	(I) Filter	(J) Filter	Mean Difference (I − J)	SE	Significance	Confidence Interval 95%	
Bonferroni	HOME	SPEAK	−31.77 *	4.69	<0.001	−44.17 to	−19.37
		SPORT	−14.77 *	4.69	0.010	−27.17 to	−2.37
		ENERGY	−21.87 *	4.69	<0.001	−34.27 to	−9.47
	SPEAK	HOME	31.77 *	4.69	<0.001	19.37 to	44.17
		SPORT	17.00 *	4.69	0.002	4.60 to	29.40
		ENERGY	9.90	4.69	0.210	−2.50 to	22.30
	SPORT	HOME	14.77 *	4.69	0.010	2.37 to	27.17
		SPEAK	−17.00 *	4.69	0.002	−29.40 to	−4.60
		ENERGY	−7.10	4.69	0.783	−19.50 to	5.30
	ENERGY	HOME	21.87 *	4.69	<0.000	9.47 to	34.27
		SPEAK	−9.90	4.69	0.210	−22.30 to	2.50
		SPORT	7.10	4.69	0.783	−5.30 to	19.50
T3 Dunnett	HOME	SPEAK	−31.77 *	4.33	<0.001	−43.23 to	−20.32
		SPORT	−14.77 *	4.40	0.005	−26.41 to	−3.13
		ENERGY	−21.87 *	4.62	<0.001	−34.09 to	−9.65
	SPEAK	HOME	31.77 *	4.33	<0.001	20.32 to	43.23
		SPORT	17.00 *	4.75	0.002	4.44 to	29.56
		ENERGY	9.90	4.96	0.247	−3.20 to	23.01
	SPORT	HOME	14.77 *	4.40	0.005	3.13 to	26.41
		SPEAK	−17.00 *	4.75	0.002	−29.56 to	−4.44
		ENERGY	−7.10	5.02	0.642	−20.36 to	6.17
	ENERGY	HOME	21.87 *	4.62	<0.001	9.65 to	34.09
		SPEAK	−9.90	4.96	0.247	−23.01 to	3.20
		SPORT	7.10	5.02	0.642	−6.17 to	20.36

* Mean difference is significant at 0.05. In bold: statistically significant values.

**Table 6:** Preliminary summary statistics relating to meters traveled.

		N	Mean	SD	SE	Middle Confidence Interval 95%		Min	Max	Components Variance
HOME		217	**471.61**	41.11	2.791	466.11 to	477.11	378	546	
SPEAK		217	**503.39**	48.85	3.326	496.85 to	509.92	378	588	
SPORT		217	**486.39**	50.18	3.406	479.67 to	493.10	378	588	
ENERGY		217	**493.48**	54.29	3.686	486.22 to	500.75	378	588	
Total		868	**488.72**	50.12	1.701	485.38 to	492.06	378	588	
Model	Fixed effects			48.84	1.658	485.46 to	491.97			
	Casual effects				6.683	467.45 to	509.99			167.644

In bold: statistically significant values.

**Table 7:** Multiple comparisons. Dependent variable: dyspnea index.

	(I) Filter	(J) Filter	Mean Difference (I − J)	SE	Significance	Confidence Interval 95%	
Bonferroni	HOME	SPEAK	0.52 *	0.15	0.005	0.11 to	0.92
		SPORT	0.29	0.15	0.360	−0.12 to	0.70
		ENERGY	1.39 *	0.15	<0.001	0.98 to	1.79
	SPEAK	HOME	−0.52 *	0.15	0.005	−0.92 to	−0.11
		SPORT	−0.23	0.15	0.860	−0.63 to	0.18
		ENERGY	0.87 *	0.15	<0.001	0.46 to	1.28
	SPORT	HOME	−0.29	0.15	0.360	−0.70 to	0.12
		SPEAK	0.23	0.15	0.860	−0.18 to	0.63
		ENERGY	1.10 *	0.15	<0.001	0.69 to	1.50
	ENERGY	HOME	−1.39 *	0.15	<0.001	−1.79 to	−0.98
		SPEAK	−0.87 *	0.15	<0.001	−1.28 to	−0.46
		SPORT	−1.10 *	0.15	<0.001	−1.50 to	−0.69
T3 Dunnett	HOME	SPEAK	0.52 *	0.15	0.005	0.11 to	0.92
		SPORT	0.29	0.15	0.306	−0.12 to	0.70
		ENERGY	1.39 *	0.16	<0.001	0.95 to	1.82
	SPEAK	HOME	−0.52 *	0.15	0.005	−0.92 to	−0.11
		SPORT	−0.23	0.14	0.525	−0.61 to	0.15
		ENERGY	0.87 *	0.15	<0.001	0.46 to	1.28
	SPORT	HOME	−0.29	0.15	0.306	−0.70 to	0.12
		SPEAK	0.23	0.14	0.525	−0.15 to	0.61
		ENERGY	1.10 *	0.15	<0.001	0.69 to	1.51
	ENERGY	HOME	−1.39 *	0.16	<0.001	−1.82 to	−0.95
		SPEAK	−0.87 *	0.155	<0.001	−1.28 to	−0.46
		SPORT	−1.10 *	0.155	<0.001	−1.51 to	−0.69

* Mean difference is significant at 0.05. In bold: statistically significant values.

**Table 8:** Preliminary summary statistics relating to dyspnea index.

		N	Mean	SD	SE	Middle Confidence Interval 95%		Min	Maz	Components Variance
HOME		217	**2.84**	1.69	0.115	2.61 to	3.06	0	7	
SPEAK		217	**2.32**	1.49	0.101	2.12 to	2.52	0	5	
SPORT		217	**2.55**	1.50	0.102	2.35 to	2.75	0	5	
ENERGY		217	**1.45**	1.72	0.122	1.22 to	1.68	0	7	
Total		868	**2.29**	1.68	0.057	2.18 to	2.40	0	7	
Model	Fixed effects			1.61	0.055	2.18 to	2.40			
	Casual effects				0.299	1.34 to	3.24			0.345

In bold: statistically significant values.

**Table 9:** Multiple comparisons. Dependent variable: final subjective fatigue index.

	(I) Filter	(J) Filter	Mean difference (I − J)	SE	Significance	Confidence Interval 95%	
Bonferroni	HOME	SPEAK	0.32	0.13	0.090	−0.03 to	0.67
		SPORT	−0.13	0.13	1.000	−0.48 to	0.22
		ENERGY	0.42 *	0.13	0.010	0.07 to	0.77
	SPEAK	HOME	−0.32	0.13	0.090	−0.67 to	0.03
		SPORT	−0.45 *	0.13	0.004	−0.80 to	−0.10
		ENERGY	0.10	0.13	1.000	−0.25 to	0.45
	SPORT	HOME	0.13	0.13	1.000	−0.22 to	0.48
		SPEAK	0.45 *	0.13	0.004	0.10 to	0.80
		ENERGY	0.55 *	0.13	<0.000	0.20 to	0.90
	ENERGY	HOME	−0.42 *	0.13	0.010	−0.77 to	−0.07
		SPEAK	−0.10	0.13	1.000	−0.45 to	0.25
		SPORT	−0.55 *	0.13	<0.001	−0.90 to	−0.20
T3 Dunnett	HOME	SPEAK	0.32	0.14	0.126	−0.05 to	0.69
		SPORT	−0.13	0.15	0.947	−0.52 to	0.27
		ENERGY	0.42 *	0.13	0.010	0.07 to	0.77
	SPEAK	HOME	−0.32	0.14	0.126	−0.69 to	0.05
		SPORT	−0.45 *	0.13	0.004	−0.80 to	−0.10
		ENERGY	0.10	0.11	0.948	−0.20 to	0.39
	SPORT	HOME	0.13	0.15	0.947	−0.27 to	0.52
		SPEAK	0.45 *	0.13	0.004	0.10 to	0.80
		ENERGY	0.55 *	0.12	<0.001	0.22 to	0.87
	ENERGY	HOME	−0.42 *	0.13	0.010	−0.77 to	−0.07
		SPEAK	−0.10	0.11	0.948	−0.39 to	0.20
		SPORT	−0.55 *	0.12	<0.001	−0.87 to	−0.22

* Mean difference is significant at 0.05. In bold: statistically significant values.

**Table 10:** Preliminary summary statistics relating to final fatigue.

		N	Mean	SD	SE	Middle Confidence Interval 95%		Min	Max	Components Variance
HOME		217	1.19	1.64	0.111	0.97 to	1.41	0.00	6.00	
SPEAK		217	0.87	1.27	0.086	0.70 to	1.04	0.00	4.00	
SPORT		217	1.32	1.47	0.100	1.13 to	1.52	0.00	4.00	
ENERGY		217	0.77	1.07	0.073	0.63 to	0.92	0.00	4.00	
Total		868	1.04	1.39	0.047	0.95 to	1.13	0.00	6.00	
Model	Fixed effects			1.38	0.047	0.95 to	1.13			
	Casual effects				0.130	0.63 to	1.45			0.059

**Table 11:** Data relations.

		Saturation	HR	Meters Traveled	Basal Dyspnea Index	Final Dyspnea Index	Basal Fatigue	Final Fatigue
Saturation	Pearson correlation	--						
	N	868						
HR	Pearson correlation	−0.185 **	--					
	Significance (two-tailed)	<0.001						
	N	868	868					
Meters traveled	Pearson correlation	0.264 **	0.248**	--				
	Significance (two-tailed)	<0.001	<0.001					
	N	868	868	868				
Basal dyspnea index	Pearson correlation	−0.259 **	0.050	−0.254 **	--			
	Significance (two-tailed)	<0.001	0.144	<0.001				
	N	868	868	868	868			
Final dyspnea index	Pearson correlation	−0.257 **	0.126 **	−0.091 **	0.408 **	--		
	Significance (two-tailed)	<0.001	<0.001	0.007	<0.001			
	N	868	868	868	868	868		
Basal fatigue	Pearson correlation	−0.014	0.024	0.047	−0.091 **	−0.018	--	
	Significance (two-tailed)	0.678	0.474	0.166	0.007	0.595		
	N	868	868	868	868	868	868	
Final fatigue	Pearson correlation	−0.077 *	−0.088 **	−0.039	−0.024	−0.187 **	0.754 **	--
	Significance (two-tailed)	0.023	0.010	0.250	0.484	<0.001	<0.001	
	N	868	868	868	868	868	868	868

** The correlation is significant at the 0.01 level (two-tailed). * The correlation is significant at the 0.05 level (two-tailed). In bold: statistically significant values.

## Data Availability

Data are contained within the article.
